# Supplementary material for: Synthesis of bio-based methylcyclopentadiene via direct hydrodeoxygenation of 3-methylcyclopent-2-enone derived from cellulose
Source: Nat Commun. 2021 Jan 4;12:46. doi: 10.1038/s41467-020-20264-3 (PMC7782798; doi:10.1038/s41467-020-20264-3)
Supplement: Supplementary file 1 — Supplementary Information [file 41467_2020_20264_MOESM1_ESM.pdf]

Supporting information

Synthesis of bio-based methylcyclopentadiene via direct hydrodeoxygenation of 3-methylcyclopent-2-enone derived from cellulose

Yanting Liu[1,2,⋆], Ran Wang[1,3,⋆], Haifeng Qi[1], Xiao Yan Liu[1], Guangyi Li[1], Aiqin Wang[1, 4], Xiaodong Wang[1], Yu Cong[1], Tao Zhang[1,2,4,*] & Ning Li[1,4,5,*]

[1] CAS Key Laboratory of Science and Technology on Applied Catalysis, Dalian Institute of Chemical Physics, Chinese Academy of Sciences, Dalian 116023, China

[2] State Key Laboratory of Catalysis, Dalian Institute of Chemical Physics, Chinese Academy of Sciences, Dalian 116023, China

[3] University of Chinese Academy of Sciences, Beijing 100049, China

[4] *i*ChEM (Collaborative Innovation Center of Chemistry for Energy Materials), Dalian Institute of Chemical Physics, Chinese Academy of Sciences, Dalian 116023, China

[5] Dalian National Laboratory for Clean Energy, Dalian 116023, China

⋆ These authors contributed equally to this work.

Correspondence and requests for materials should be addressed to to N. L. (email: lining@dicp.ac.cn) or T. Z. (email: taozhang@dicp.ac.cn)

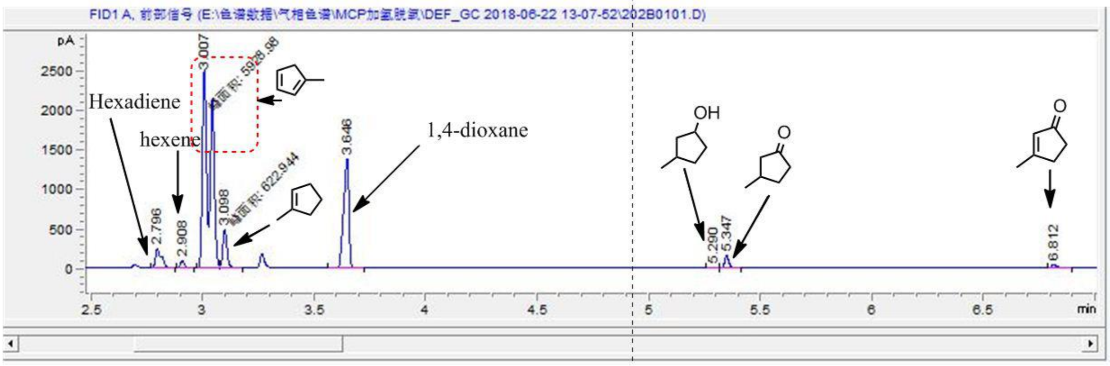

Supplementary Figure 1. GC chromatogram of the product obtained from the hydrodeoxygenation of MCP over the 15wt.%MoO_3/ZnO catalyst.

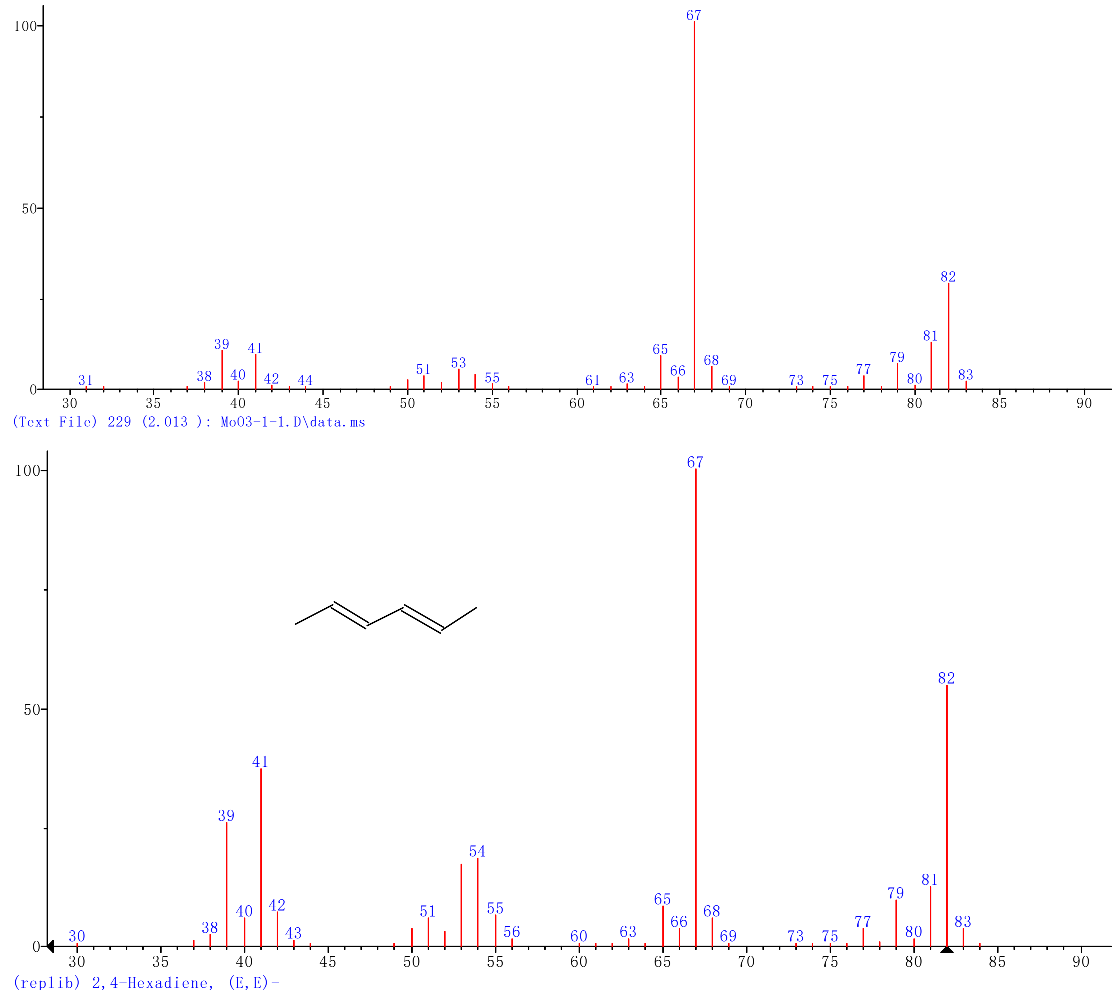

Supplementary Figure 2. Mass spectrograms of product hexadiene (HDE).

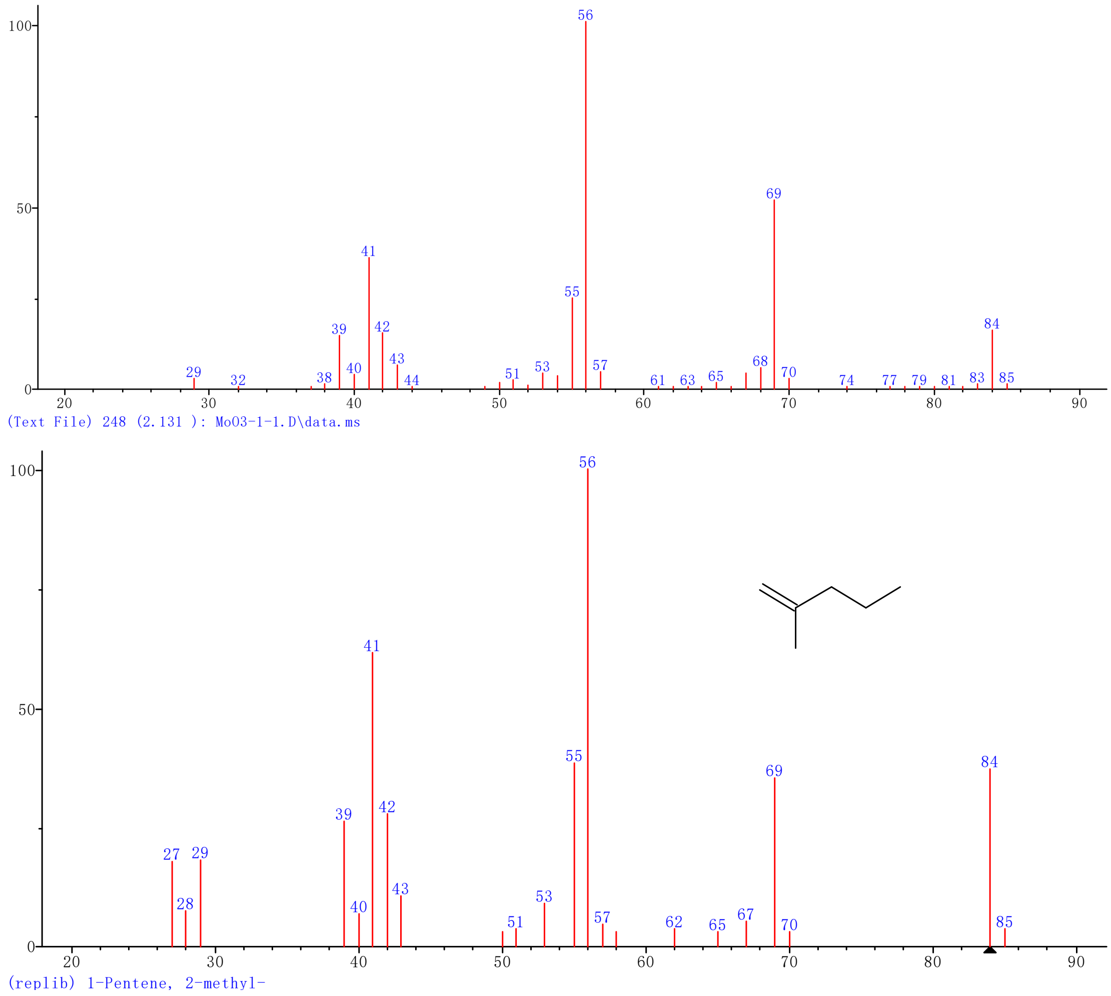

Supplementary Figure 3. Mass spectrograms of product hexene (HE).

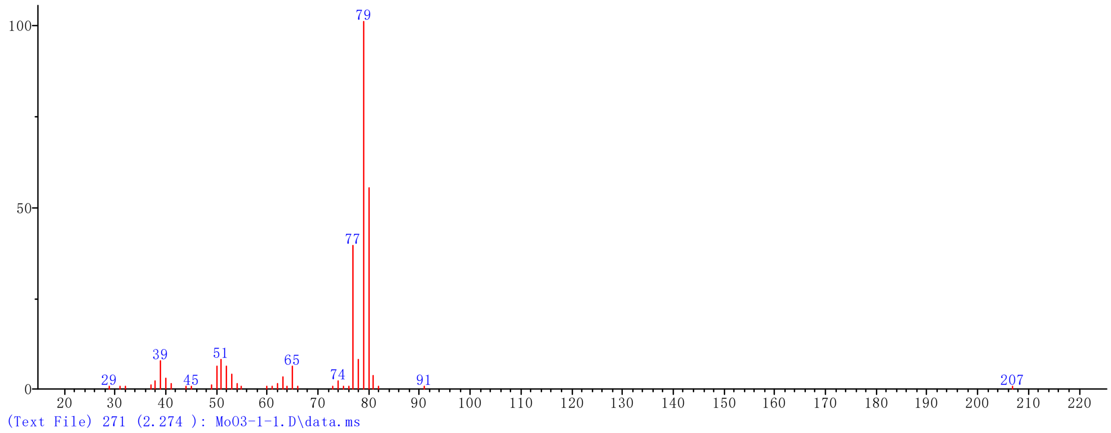

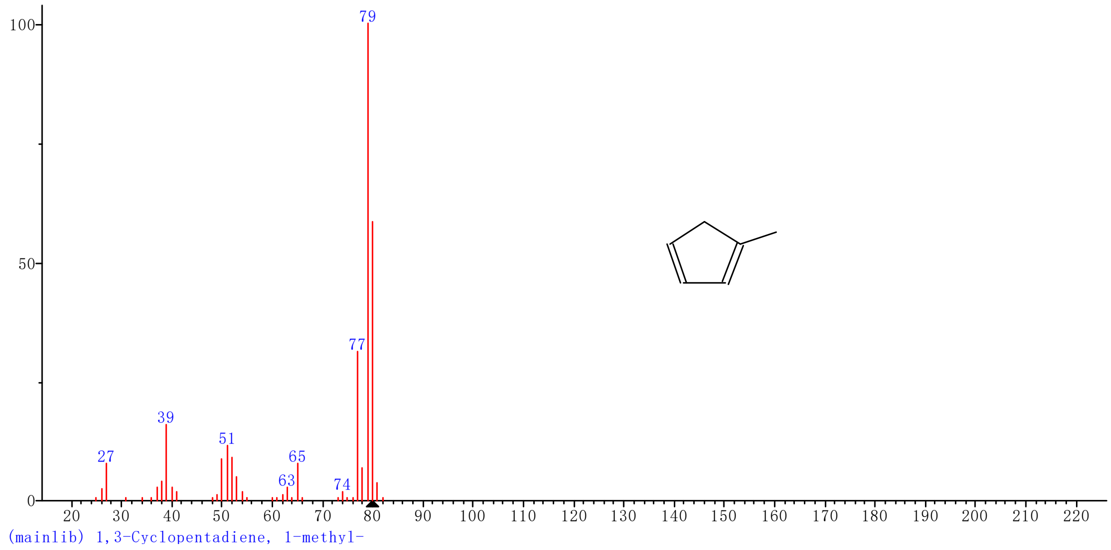

Supplementary Figure 4. Mass spectrograms of product methylcyclopentadiene (MCPD).

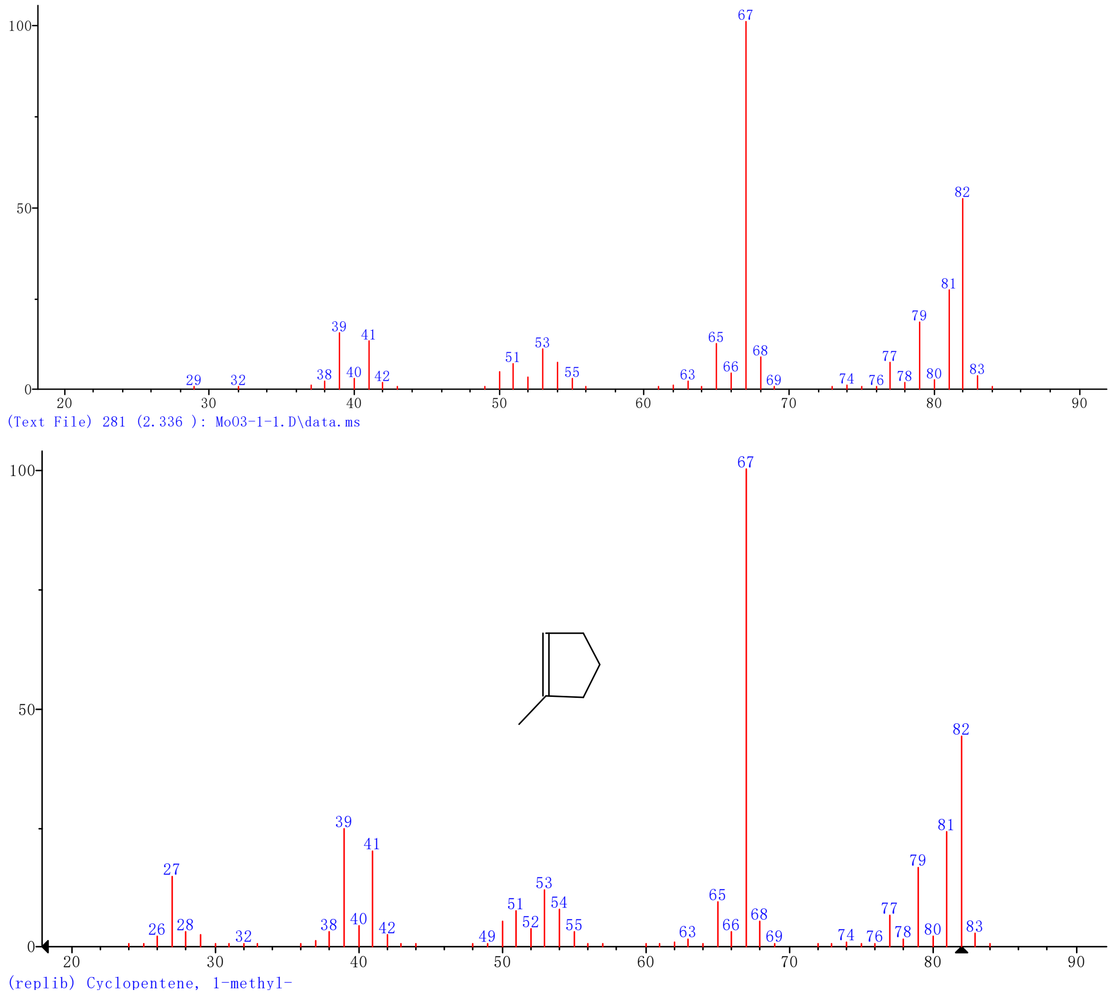

Supplementary Figure 5. Mass spectrograms of product methylcyclopentene (MCPE).

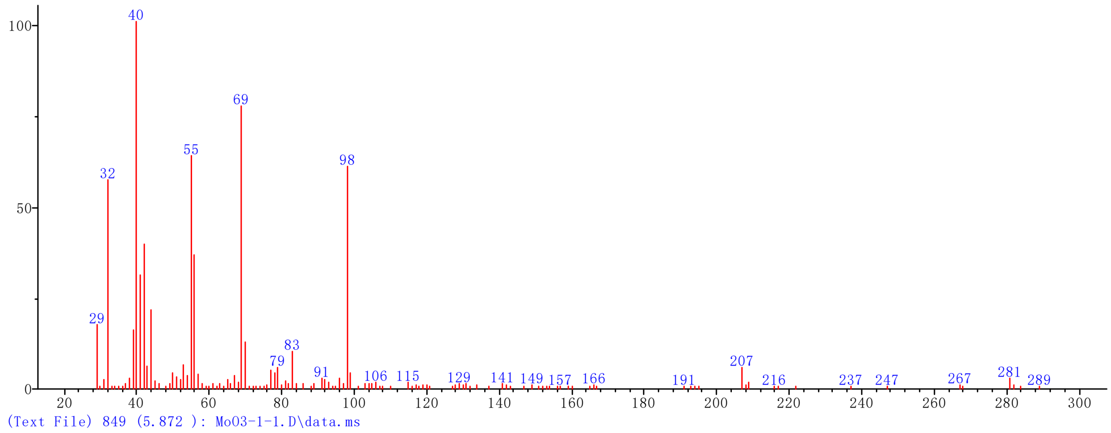

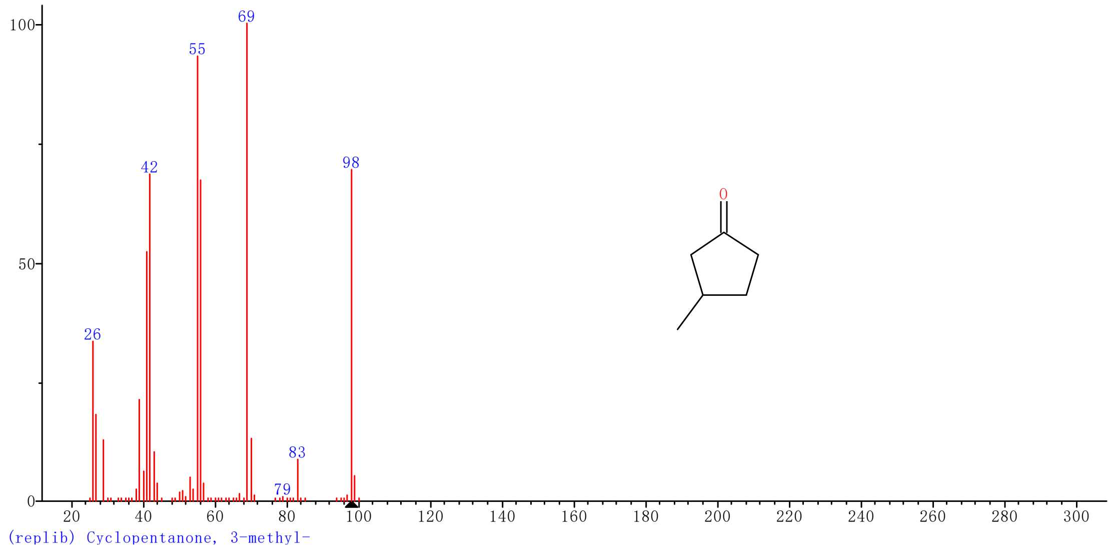

Supplementary Figure 6. Mass spectrograms of product 3-methylcyclopentanone (MCPO).

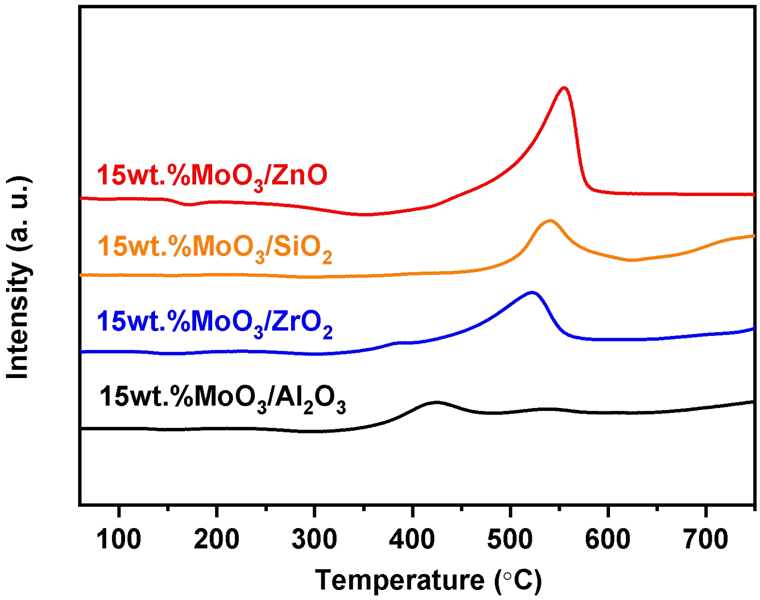

Supplementary Figure 7. H_2-TPR profiles of the supported molybdenum oxide catalysts.

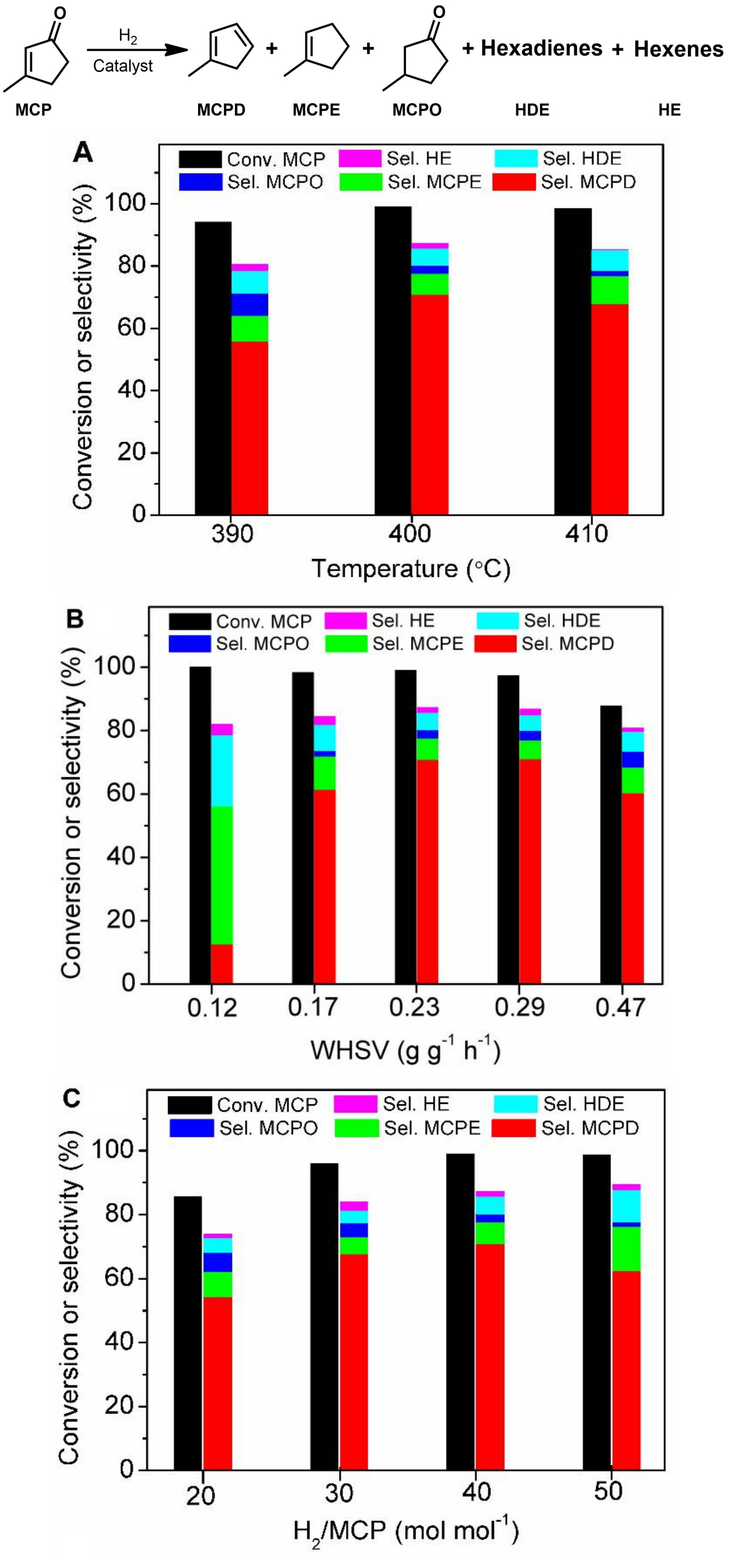

Supplementary Figure 8. The performances of the 15wt.%MoO_3/ZnO catalyst for the hydrodeoxygenation of MCP as the functions of (A) reaction temperature, (B) weight hourly space velocity (WHSV) and (C) initial molar ratio of H_2/MCP. Reaction conditions: (A) P_{H_2} = 0.1 MPa, WHSV = 0.23 (g g^{-1} h^{-1}), initial H_2/MCP molar ratio = 40; (B) T = 400 °C, P_{H_2} = 0.1 MPa, initial H_2/MCP molar ratio = 40; (C) T = 400 °C, P_{H_2} = 0.1 MPa, WHSV = 0.23 (g g^{-1} h^{-1}).

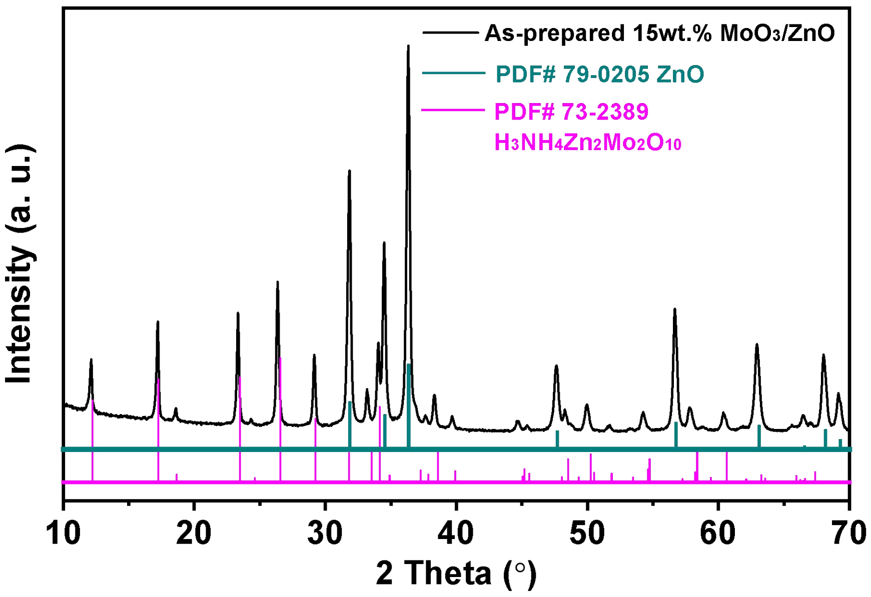

Supplementary Figure 9. XRD patterns of 15wt.%MoO_3/ZnO sample prepared by the impregnation of ZnO with $(NH_4)_6Mo_7O_{24}\cdot 4H_2O$ solution.

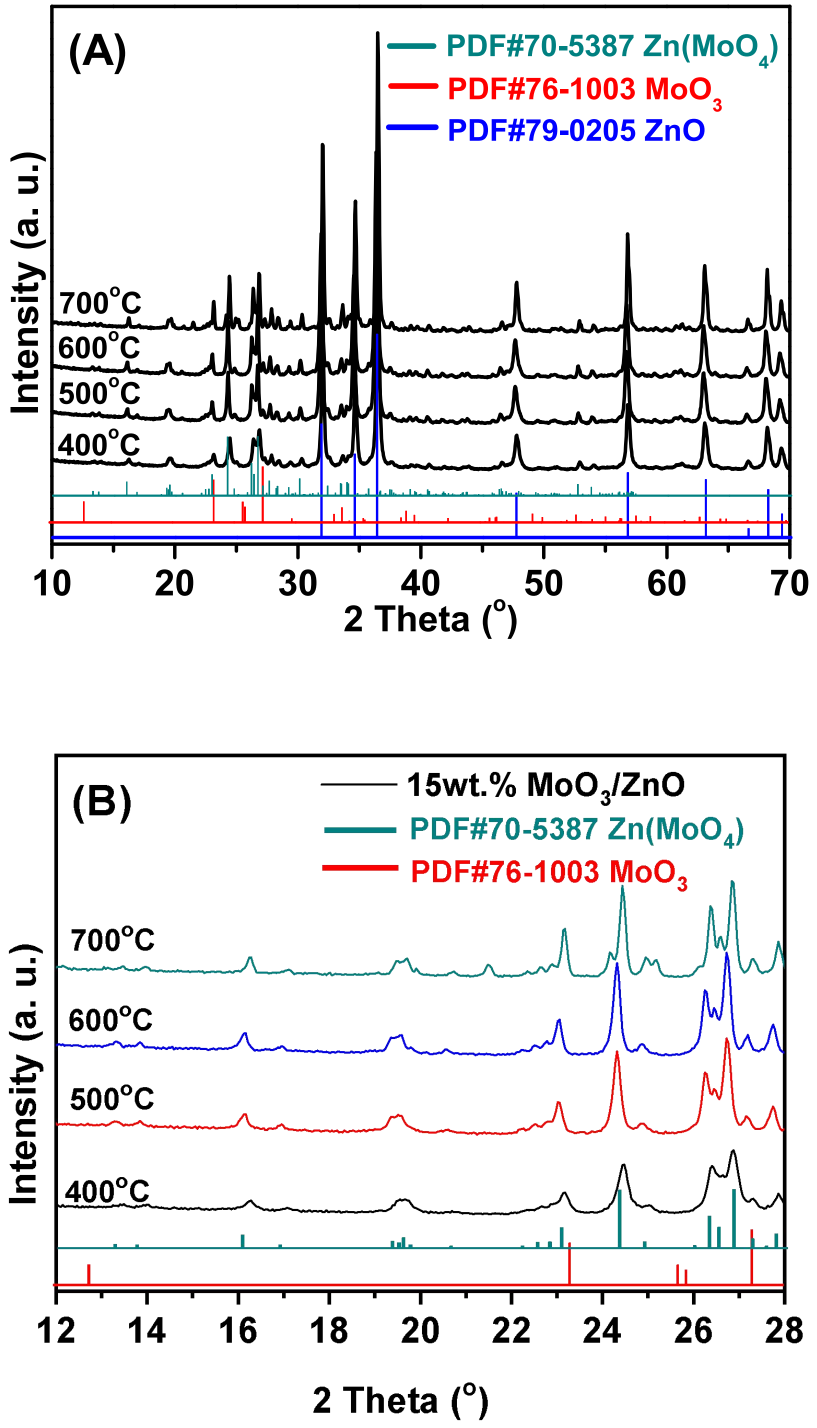

Supplementary Figure 10. XRD patterns of (A) 15wt.%MoO_3/ZnO samples after being calcined at different temperatures. (B) The enlargement of the corresponding area in (A).

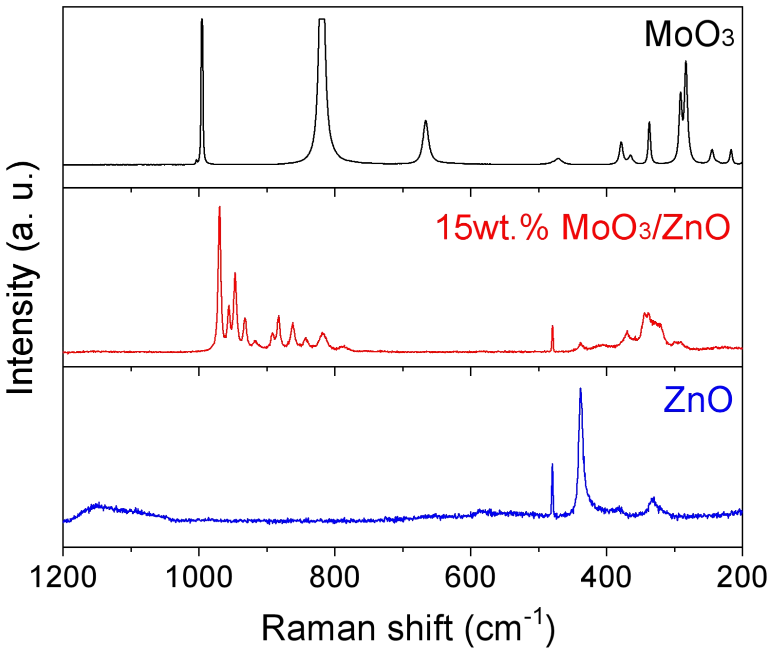

Supplementary Figure 11. Raman spectra of the calcined MoO_3, 15wt.%MoO_3/ZnO and ZnO catalysts.

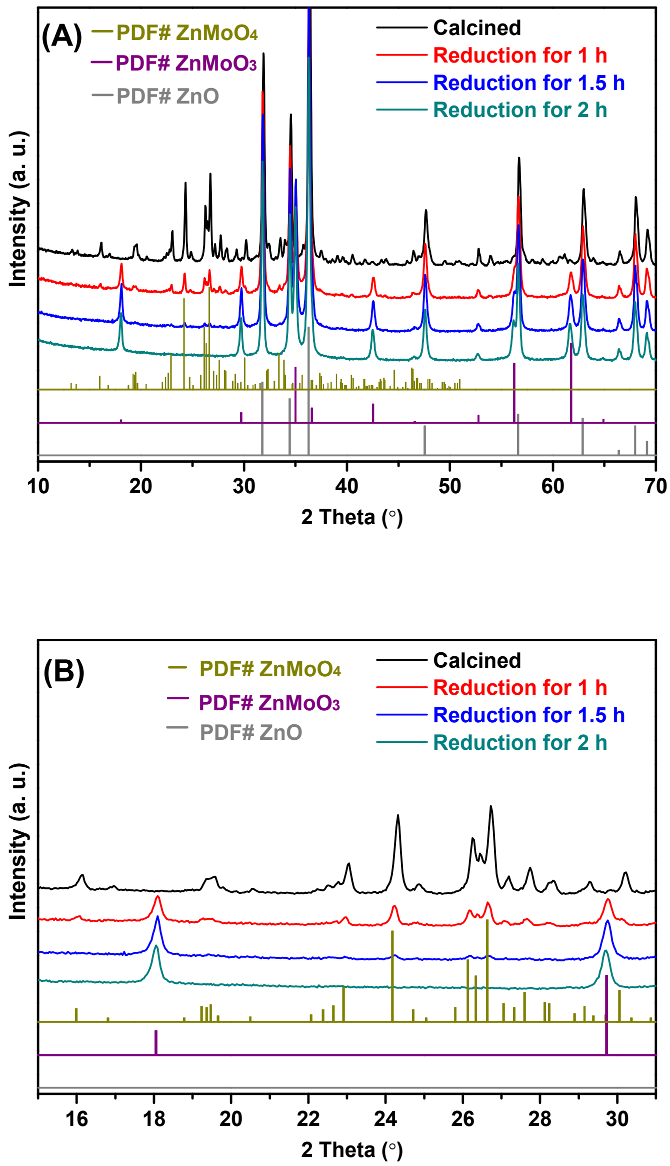

Supplementary Figure 12. XRD patterns of (A) the calcined and reduced 15wt.%MoO_3/ZnO samples. (B) The enlargement of the corresponding area in (A).

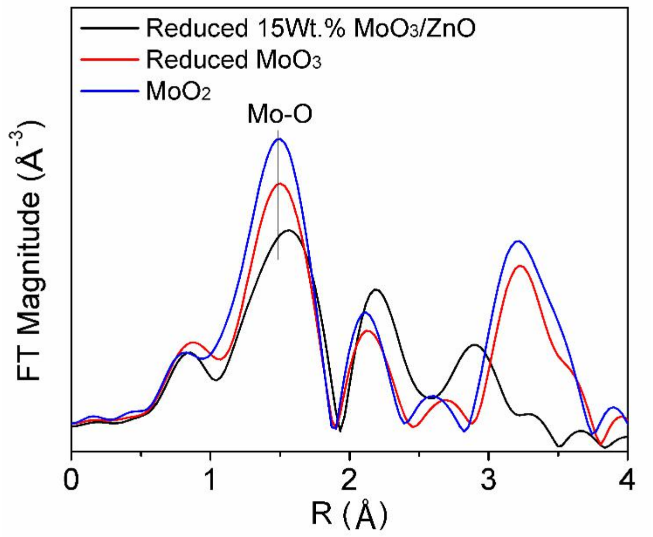

Supplementary Figure 13. The Fourier transform (FT) of k^2-weighted EXAFS spectra at the K-edge of Mo of the reduced MoO_3, 15wt.%MoO_3/ZnO catalyst and the reference MoO_2.

Supplementary Table 1. EXAFS data fitting results at the K-edge of Mo of the reduced MoO_3, 15wt.%MoO_3/ZnO catalyst and the reference MoO_2.

Sample	Shell	N[a]	R (Å)[b]	σ^2 ($\times 10^{-2}$ Å^2)[c]	ΔE_0 (eV)[d]	r-factor (%)
MoO_2	Mo-O	6.0	2.02	0.33	-1.7	0.9
15wt.%MoO_3/ZnO-reduced	Mo-O	4.3	2.06	0.94	3.6	0.9
MoO_3- reduced	Mo-O	4.8	2.01	0.50	1.1	0.9

[a] N, the coordination number for the absorber-backscatterer pair.

[b] R, the average absorber−backscatterer distance.

[c] σ^2, the Debye−Waller factor.

[d] ΔE_0, the inner potential correction. The accuracies of the above parameters were estimated as N, ±20%; R, ±1%; σ^2, ±20%; ΔE_0, ±20%. The Δk (2.8 – 13.4 Å^{-1}) and ΔR (1.0 – 1.9 Å) are the data ranges used for data fitting in k-space and R-space, respectively.

Supplementary Table 2. Physical properties of zinc-molybdenum oxide catalysts.

Entry	Catalyst	$T_{calcination}$ (°C)	S_{BET} (m^2 g^{-1})[a]	D_{pore} (nm)[a]	V_{pore} (mL g^{-1})[a]
1	10wt.%MoO_3/ZnO	600	30.5	3.4	0.1
2	15wt.%MoO_3/ZnO	600	19.2	3.4	0.1
3	20wt.%MoO_3/ZnO	600	14.3	3.4	0.1
4	15wt.%MoO_3/ZnO	400	15.7	29.9	0.2
5	15wt.%MoO_3/ZnO	500	12.0	30.3	0.1
6	15wt.%MoO_3/ZnO	700	6.1	3.0	0.0

[a] Specific BET surface areas (S_{BET}), average pore sizes (D_{pore}) and average pore volumes (V_{pore}) of the zinc-molybdenum oxide catalysts were measured by N_2-physisorption.

Supplementary Table 3. The aldol condensation of HD to MCP over the 15wt.%MoO_3/ZnO catalyst.[a]

Conversion of HD (%)	S_{MCP} (%)[b]	S_{DMF} (%)[b]	S_{Others} (%)[b]	Yield of MCP (%)
53.5	70.5	8.6	20.9	37.8

[a]Reaction conditions: T = 400 °C, P_{N_2} = 0.1 MPa, weight hour space velocity (WHSV) = 0.23 (g g^{-1} h^{-1}), the initial N_2/HD molar ratio = 40. HD: 2,5-hexanedione, MCP: 3-methylcyclopent-2-enone. [b]S_{MCP}: Selectivity of MCP, S_{DMF}: Selectivity of 2,5-dimethylfuran, S_{Others}: Selectivity of the other by-products generated during the reaction (include C_{12} oligomers and coke).

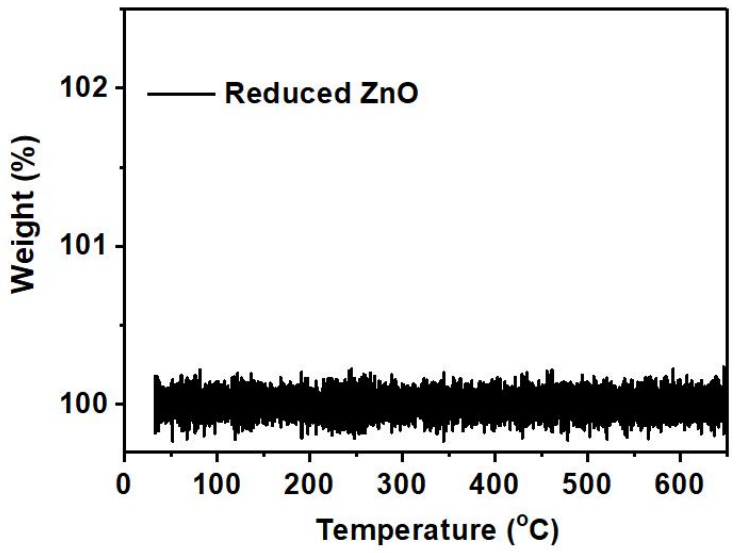

Supplementary Figure 14. TG profiles of the ZnO support which was reduced at 400 °C for 2 h.

Calculation method for the oxygen vacancy concentrations of the catalysts:

On the one hand, the mass fraction of Mo in MoO_3 is 0.67 (*i.e.* the relative atomic weight of Mo / the relative molecular weight of MoO_3 = 96 / 144 = 0.67). The oxygen vacancy concentrations of MoO_3 catalyst is 1.988%. Therefore, we can calculate the oxygen vacancy concentrations of the catalyst based on per gram Mo, *i.e.*, 1.988% / 0.67 = 2.982%.

On the other hand, the Mo content of 15wt.%MoO_3/ZnO is 0.15. The oxygen vacancy concentrations of 15wt.%MoO_3/ZnO catalyst is 2.261%. Therefore, we can calculate the oxygen vacancy concentrations of the catalyst based on per gram Mo, *i.e.*, 2.261% / 0.15 = 15.073%.

Based on the above information, the oxygen vacancy concentrations (based on per gram Mo of catalyst) of the 15wt.%MoO_3/ZnO catalyst is 5.055 (obtained by 15.073% / 2.982%) times that of MoO_3 catalyst.

Finally, when the oxygen vacancy concentrations of MoO_3 catalyst is 1.988%, corresponding the oxygen vacancy concentrations of 15wt.%MoO_3/ZnO catalyst should be 1.988% × 5.055 = 10.049%.

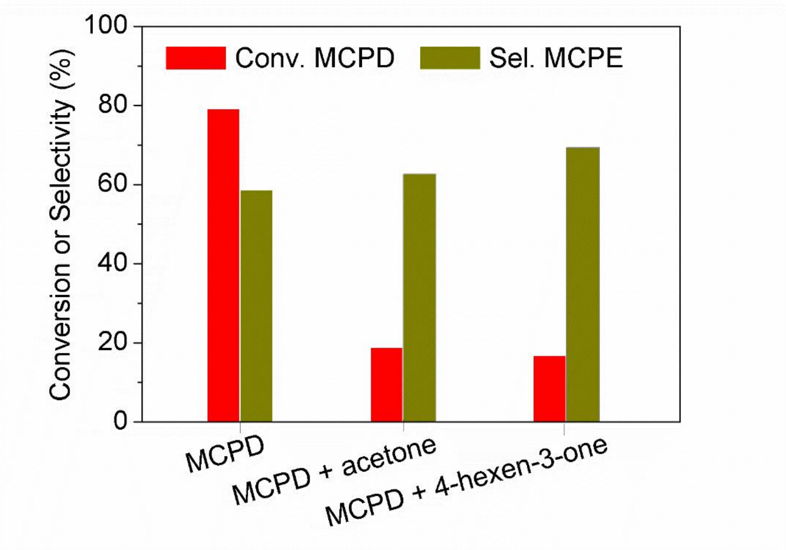

Supplementary Figure 15. The hydrogenation of MCPD, MCPD + acetone, and MCPD + 4-hexen-3-one over the 15wt.%MoO_3/ZnO catalysts. Conditions: T = 400 °C, P_{H_2} = 0.1 MPa, WHSV = 0.23 g g^{-1} h^{-1}, initial H_2/MCPD molar ratio is 40. MCPD: methylcyclopentadiene. MCPE: methylcyclopentene.

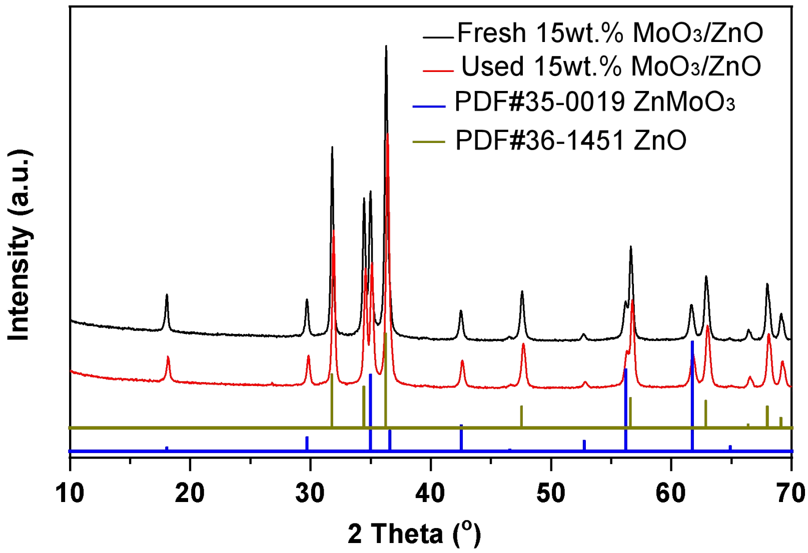

Supplementary Figure 16. XRD patterns of the fresh reduced and used 15wt.%MoO_3/ZnO catalysts.

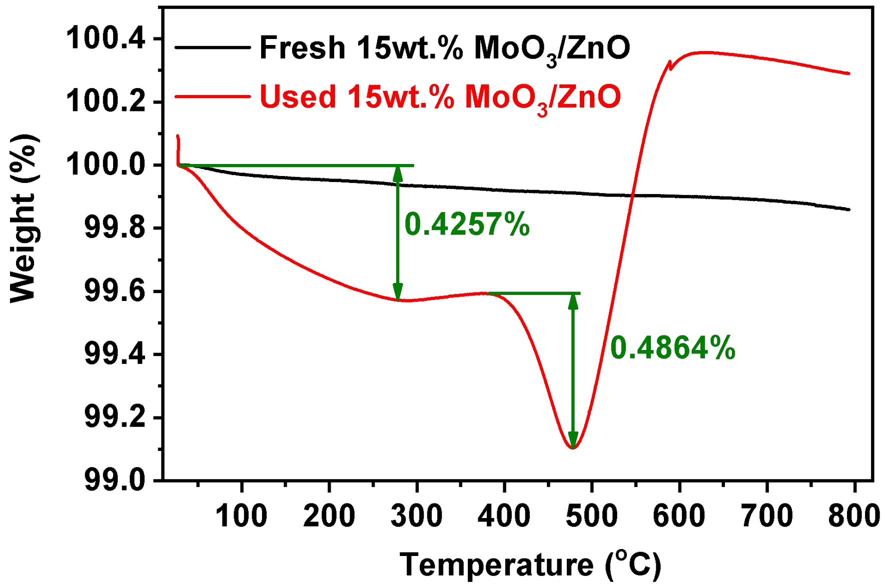

Supplementary Figure 17. TG curves of the fresh and used 15wt.%MoO_3/ZnO catalysts (the reaction conditions of used catalyst: T = 400 °C, P_{H_2} = 0.1 MPa, WHSV = 0.23 g g^{-1} h^{-1}, initial H_2/MCPD molar ratio = 40, time on stream = 20 h).

In the TG curve, the fresh 15wt.%MoO_3/ZnO catalyst exhibits a slight weight loss at full temperature region (30−800 °C), while the used 15wt.%MoO_3/ZnO catalyst gives a significant weight loss at a high temperature region (380-500 °C). The weight loss at low temperature may be attributed to the desorption of water and reactant (or by-products), while the weight loss at the high temperature region should be related to the oxidation of coke formed on the catalyst. At higher temperature (> 500 °C), the increasing of total weight for used 15wt.%MoO_3/ZnO can be attributed to the oxidation of reduced 15wt.%MoO_3/ZnO.

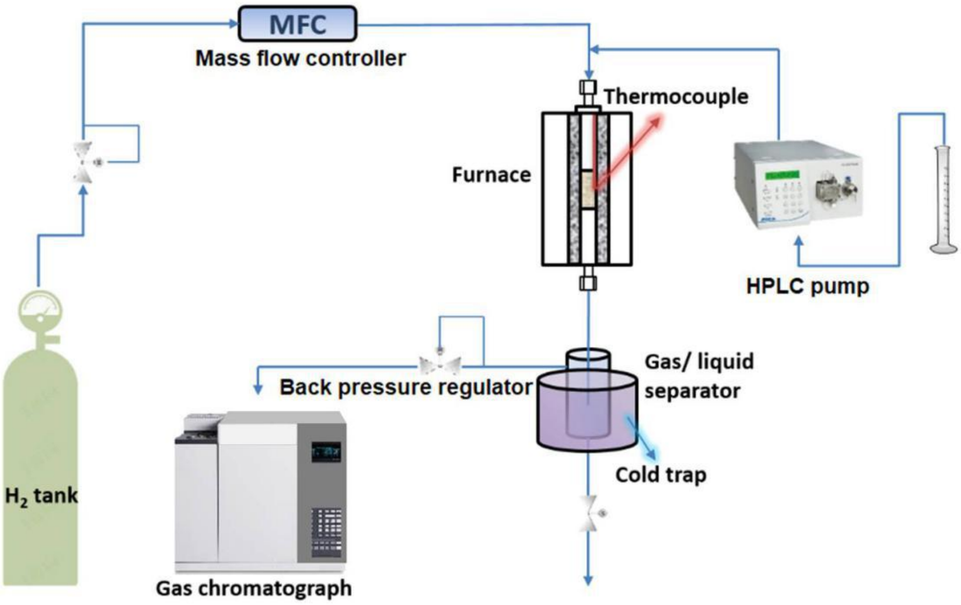

Supplementary Figure 18. Diagram of the continuous flow reactor used in MCP hydrodeoxygenation.

In each test, a certain amount of catalyst was put into the tubular reactor. To keep the catalyst located at the constant temperature area, both top and end of the reactor were filled with quartz sand and quartz wool. After the system pressure and temperature are stabilized, the liquid reactant was pumped into the system at a certain flow rate using a high-pressure liquid chromatography pump (HPLC), accompanied with the hydrogen. The hydrogen flow rate was controlled by a mass flow controller (MFC). After coming out from the reactor and being cooled down in a trap, the product was divided into two phases through a gas-liquid separator. The gas phase products were analyzed online though a gas chromatography (GC) after passing through the back pressure regulator. According to the concentration of feed (or specific compound) in the gas phase effluent products (measured with the on-line GC by external standard method), the gas flowrate of the effluent gas and reaction time, we can calculate the mole amount of feed (or specific compound) in gas phase products. The liquid product was taken out through the sampling valve, and then analyzed by another GC using 1,4-dioxane as the internal standard. According to the analysis results, we can calculate the mole amount of feed (or specific compound) detected in the liquid phase products. Thus, the conversion and selectivity of the reactions were calculated based on the analysis of gas phase products and liquid phase products according the following equations:

Conversion (%) = 100 – total mole amount of feed detected in gas phase and liquid phase products / mole amount of feed pumped into reactor × 100

Selectivity for a specific compound (%) = total mole amount of a specific compound detected in gas phase and liquid phase products / total mole amount of feed converted × 100

Based on our analysis, the carbon balances of this work are good. In most of cases, the sum of selectivity of identified products are higher than 80%.

In the reaction system, the packing volume of 2.5 g catalyst is 1.6 mL. The initial H_2/MCP molar ratio used in MCP hydrodeoxygenation is 40. The reaction temperature (400 ^oC) is higher than the boiling point of MCP (157.5 ^oC). Therefore, when H_2 flow rate is 90 mL min^{-1}, the flow rate of the MCP after being vaporized in tubular reactor is 2.25 (*i. e.*, 90 / 40) mL min^{-1}. The residence time (t) of MCP on the catalyst is t = the packing volume of catalyst / the flow rate of MCP after being vaporized = 1.6 mL / 2.25 mL min^{-1} = 0.711 min = 42.67 s.
